# Assessing the Impact of Environmental and Management Variables on Mountain Meadow Yield and Feed Quality Using a Random Forest Model

**DOI:** 10.3390/plants14142150

**Published:** 2025-07-11

**Authors:** Adrián Jarne, Asunción Usón, Ramón Reiné

**Affiliations:** Departamento de Ciencias Agrarias y del Medio Natural, Escuela Politécnica Superior, Universidad de Zaragoza, Ctra Cuarte s/n, 22071 Huesca, Spain; mauson@unizar.es (A.U.); rreine@unizar.es (R.R.)

**Keywords:** grassland, protein content, relative feed value, cutting date, modeling

## Abstract

Seasonal climate variability and agronomic management profoundly influence both the productivity and nutritive value of temperate hay meadows. We analyzed five years of data (2019, 2020, 2022–2024) from 15 meadows in the central Spanish Pyrenees to quantify how environmental variables (January–June minimum temperatures, rainfall), management variables (fertilization rates (N, P, K), livestock load, cutting date), and vegetation (plant biodiversity (Shannon index)) drive total biomass yield (kg ha^−1^), protein content (%), and Relative Feed Value (RFV). Using Random Forest regression with rigorous cross-validation, our yield model achieved an R^2^ of 0.802 (RMSE = 983.8 kg ha^−1^), the protein model an R^2^ of 0.786 (RMSE = 1.71%), and the RFV model an R^2^ of 0.718 (RMSE = 13.86). Variable importance analyses revealed that March rainfall was the dominant predictor of yield (importance = 0.430), reflecting the critical role of early-spring moisture in tiller establishment and canopy development. In contrast, cutting date exerted the greatest influence on protein (importance = 0.366) and RFV (importance = 0.344), underscoring the sensitivity of forage quality to harvest timing. Lower minimum temperatures—particularly in March and May—and moderate livestock densities (up to 1 LU) were also positively associated with enhanced protein and RFV, whereas higher biodiversity (Shannon ≥ 3) produced modest gains in feed quality without substantial yield penalties. These findings suggest that adaptive management—prioritizing soil moisture conservation in early spring, timely harvesting, balanced grazing intensity, and maintenance of plant diversity—can optimize both the quantity and quality of hay meadow biomass under variable climatic conditions.

## 1. Introduction

Mountain meadows are crucial ecosystems in mountainous regions, vital for both ecological balance and agricultural sustainability [1,2,3]. These diverse environments support a rich variety of plant and animal life, acting as biodiversity hotspots [4,5,6]. In addition to provisioning, they provide essential ecosystem services, including water storage and regulation from snowmelt, which replenishes groundwater and maintains summer stream flows [7,8,9]. The dense vegetation and organic soils filter pollutants, ensuring high water quality downstream, and act as significant carbon sinks, helping to mitigate climate change [1,10,11,12].

In regions like the central Spanish Pyrenees, mountain meadows are particularly important for their natural diversity and scenic landscapes, shaped by both environmental conditions and traditional agricultural practices [13,14,15]. Agriculturally, these meadows are a primary feed source for livestock, providing hay for winter, which is essential for the economic and cultural fabric of mountain communities [16,17]. The yield and nutritional quality of the forage directly impact animal health and productivity, making their optimization a key concern for farmers [6].

The productivity and feed quality of mountain meadows are influenced by a complex interplay of environmental and management factors [18,19]. Climatic conditions, particularly temperature and precipitation, significantly affect plant growth, species composition, and nutrient cycling, making these ecosystems sensitive to climate change [11,20,21]. Agricultural management practices, such as fertilization, grazing intensity, and the timing of hay harvesting, also have profound effects on soil properties, plant communities, and the nutritional value of the forage [22,23,24,25]. For instance, fertilization can increase yield but may alter plant diversity, while grazing can impact plant species abundance and soil compaction [26]. The timing of hay cutting involves a trade-off between maximizing biomass and retaining nutritional quality [13,24]. Furthermore, the diversity of plant species within the meadows contributes to their ecological stability, resilience to environmental stresses, and overall forage quality [1].

Despite existing research on these individual factors, a comprehensive understanding of their combined and potentially non-linear impacts on forage yield and feed quality is still lacking. Integrated research approaches are necessary to simultaneously consider the complex interactions between climate, management practices, and biodiversity to achieve a more holistic understanding of these ecosystems [27]. Machine learning techniques, such as Random Forest modeling, offer a powerful framework for analyzing such complex datasets and predicting forage yield and feed quality [28,29]. Accurately predicting yield and understanding the factors influencing nutritional quality are equally important for effective livestock management and ensuring animal health and productivity [30,31]. Developing and applying models that can simultaneously evaluate the impact of these multifaceted variables will provide a more comprehensive understanding of the overall value of mountain meadows for livestock farming, allowing for more informed management decisions and sustainable practices.

## 2. Results

After collecting data over the years 2019, 2020, 2022, 2023, and 2024, the analysis has yielded the following results.

### 2.1. Data Results

The following figure displays the vegetation characteristics and biodiversity of the studied meadows.

**Figure 1:**
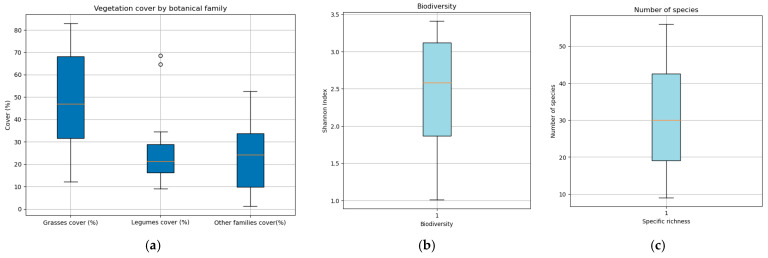
This charts summarize meadow vegetation characteristics: quartiles, median, and outliers of (**a**) vegetation cover by botanical family; (**b**) biodiversity; (**c**) specific richness.

Figure 1 quantitatively summarizes the key vegetation attributes observed in the study meadows. The data reveal that grasses overwhelmingly dominate the plant community, with cover values ranging from 82% at their highest to 33% at their lowest. The most frequent and abundant are *Dactylis glomerata*, *Poa pratensis*, *Trisetum flavescens*, *Festuca arundinacea*, and *Arrhenatherum elatius*. Legumes emerge as the second most prominent plant family, exhibiting cover percentages between 68% and 9%. The most frequent and abundant are *Trifolium pratense*, *Trifolium repens*, *Lotus corniculatus*, *Vicia cracca*, *Medicago lupulina*, and *Lathyrus pratensis*. In contrast, the collective contribution of other plant families is considerably lower, varying from 52% down to just 1%. Abundant species in this group were, among others, *Taraxacum officinale*, *Chaerophillum aureum*, *Achillea millefolium*, *Plantago lanceolata*, and *Scabiosa columbaria*.

In terms of species richness, the meadows are characterized by a high diversity, with the number of species ranging from a minimum of 9 to as many as 56. Moreover, the Shannon diversity index further highlights the elevated biodiversity across these sites, with values spanning from 3.4 in the richest communities to 1 in those with lower diversity. With the exception of four meadows that had been sown in the last 10 years, most of the floristic inventories of the meadows are attributed to the association *Rhinantho mediterranei Trisetum flavescentis* included in the *Arrhenatherion* alliance. A total of 118 species belonging to 32 families were identified.

Table 1 provides a comprehensive summary of the key factors influencing meadow performance. It details the climatic variables (such as average, maximum, and minimum temperature and rain), the management practices adopted (including cutting dates, grazing intensity, and fertilization rate), as well as the yield and quality parameters (crude protein and relative feed value).

The analysis of 15 meadows over five years (2019, 2020, 2022, 2023, and 2024) revealed substantial interannual variability in both climatic conditions and agronomic practices during the critical growing period of January to June. Our results indicate considerable fluctuations in key environmental parameters, with average January temperatures ranging from 1.64 °C in 2023 to 3.92 °C in 2024, and corresponding variations in rainfall that suggest differences in early-season moisture regimes. These climatic dynamics were evaluated in tandem with detailed management data—including fertilization (expressed as N, P, K kg ha^−1^ rates) and livestock load—which together influenced cutting dates, total biomass yield (kg ha^−1^), protein content, and RFVs.

### 2.2. Random Forest Models

#### 2.2.1. Yield Model

**Figure 2:**
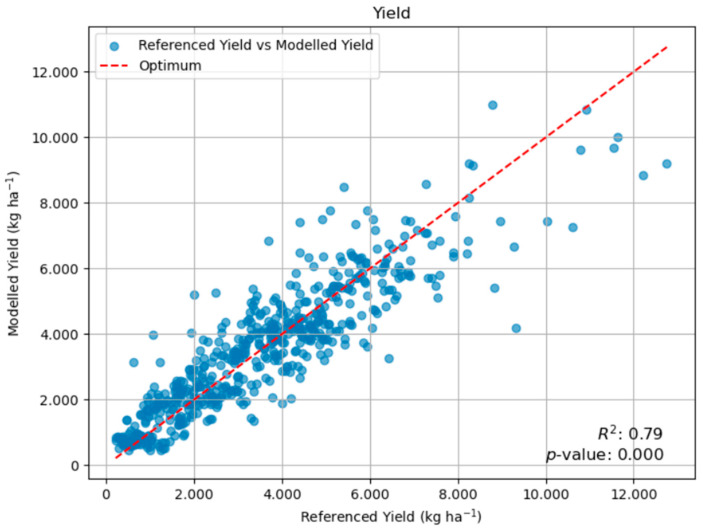
Random Forest model for yield.

In our study, we applied a Random Forest regression algorithm to predict forage yield by integrating multifactorial inputs, including climate variables, management practices, and quality parameters. This ensemble method constructs numerous decision trees from bootstrapped samples and introduces the feature of randomness at each split, which enhances its ability to capture complex, non-linear relationships among predictors. By employing cross-validation, we ensured that the model’s performance was robust and not overly reliant on any specific data partition, as we can observe in Figure 2. The resulting metrics—an RMSE of 983.84 kg/ha and an R^2^ of 0.802—highlight the model’s high predictive accuracy, with nearly 80% of the variance in yield being explained.

#### 2.2.2. Protein Model

**Figure 3:**
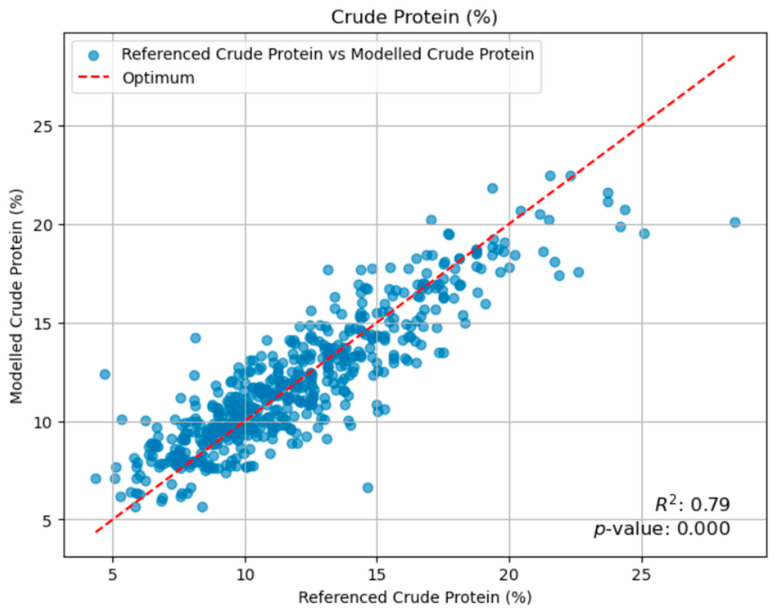
Random Forest model for crude protein content.

A Random Forest regression model was developed to predict the protein content of meadow biomass by integrating multifactorial inputs such as climate variables, fertilization rates, livestock load, and other management parameters. Figure 3 shows that there is a strong agreement between the sampling points and the model line. Through rigorous cross-validation, the model demonstrated robust predictive performance with an RMSE of 1.71 and an R^2^ of 0.786, indicating that nearly 79% of the variability in protein content could be explained by the combined effect of the studied factors.

#### 2.2.3. RFV Model

**Figure 4:**
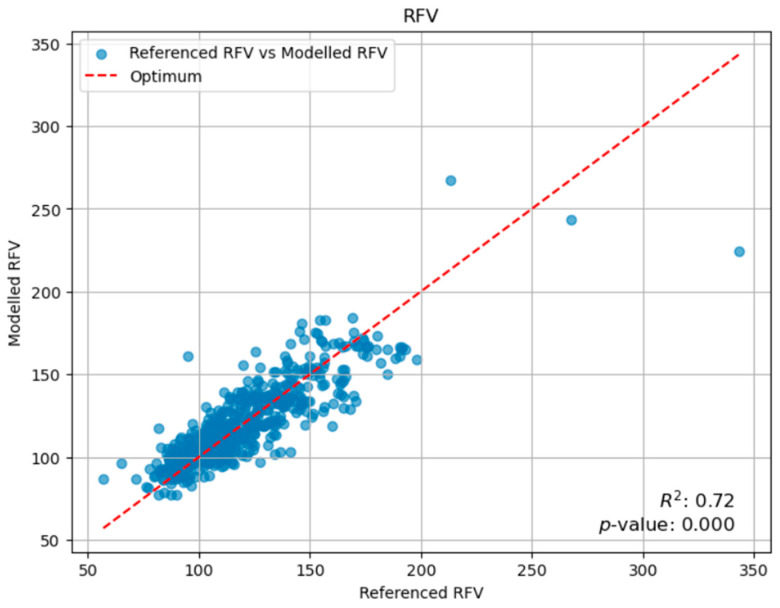
Random Forest model for Relative Feed Value (RFV).

A Random Forest regression model was developed to predict relative feed value (RFV) by integrating multifactorial inputs such as climate data, fertilization rates, and livestock load. Figure 4 illustrates a good agreement between the sampling points and the model line. Employing cross-validation to enhance robustness, the model achieved a Root Mean Square Error (RMSE) of 13.86 and a coefficient of determination (R^2^) of 0.718, indicating that nearly 72% of the variance in RFV was explained by the selected variables.

Table 2 details the importance for each of the three Random Forest models. This study uses the mean decrease in impurity (MDI) to quantify the contribution of each feature to the model’s predictive accuracy. The importance values are normalized so that the sum of all importances for each model equals one. To draw attention to the most influential predictors, values greater than 0.1 are highlighted. A value of 0.1 signifies that a variable accounts for at least 10% of the model’s predictive power, establishing it as a key driver in the model.

A standout finding is the exceptional importance of March rain in the yield model, where it registers an importance value of 0.430. This high value indicates that early spring precipitation is a major driver of biomass accumulation, likely because the rain in March supplies the essential moisture needed during the critical initial growth stages of the meadow. Such early water availability can significantly boost plant vigor and lead to enhanced yield outcomes, underscoring the pivotal role that timely rainfall plays in the overall productivity of the system.

In contrast, the cutting date emerges as a dominant factor in predicting forage quality, particularly for protein content and RFV. The model assigns relatively high importance values for the cutting date in these cases (with values around 0.366 for protein and 0.344 for RFV), which highlights its critical role in determining key nutritional attributes of the forage. The cutting date reflects a management decision that directly affects the duration of plant growth and the stage at which the meadow is harvested. An optimally timed cut can capture the peak protein accumulation in the biomass while also ensuring that the forage maintains an ideal structure for digestibility and overall feed value.

For yield predictions, although the cutting date is still an influential factor, its importance is noticeably less (0.194) compared to that of March rain. This suggests that while the timing of harvest can modulate total biomass production, it plays a secondary role to the naturally occurring climatic conditions that favor early growth. Thus, in systems where early-season moisture is abundant, yield benefits tend to accrue regardless of slight shifts in the harvest time, whereas forage quality parameters respond more sensitively to the precise timing of cutting.

**Figure 5:**
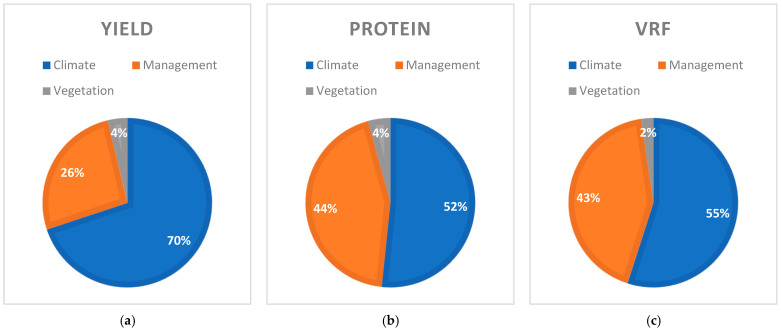
This charts summarizes the variable importance in each model: (**a**) variable importance in the yield model; (**b**) variable importance in the protein content model; (**c**) variable importance in the RFV model.

Figure 5 encapsulate a multifaceted narrative: in sustainable meadow systems, climate predominantly drives biomass production, whereas forage quality metrics such as protein content and RFV are much more sensitive to meadow management. The relatively lower importance of meadow characteristics further underscores that—even though inherent site differences exist—adaptive management can substantially optimize both productivity and nutritional outcomes.

### 2.3. Variable Impact

#### 2.3.1. Yield Model Variable Impact

The impact of the most significant variables in the model has been analyzed. In the case of the performance model, precipitation and cutting date have been examined as primary factors. Additionally, although biodiversity does not exert a strong influence on performance, it has been included due to its crucial role in conservation efforts.

**Figure 6:**
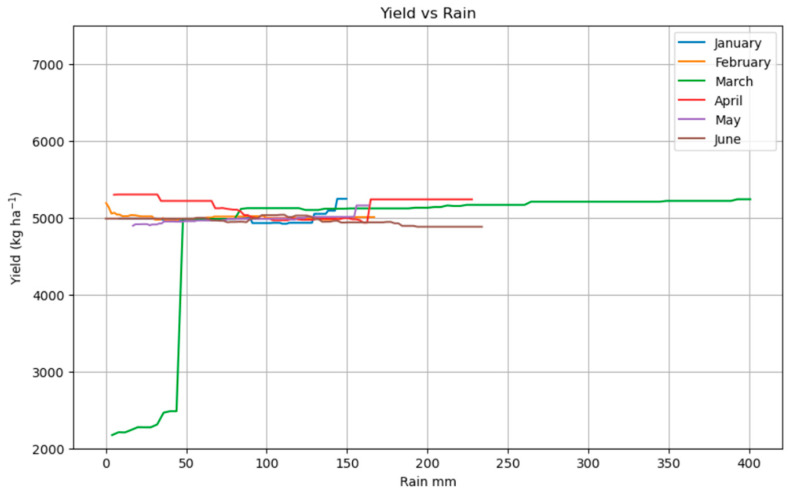
Partial dependence plot showing the impact of monthly precipitation in yield.

Figure 6 shows how the yield of a meadow (in kg/ha^−1^) responds to monthly rainfall (in mm) from January to June. For most months (January to June), the yield remains relatively stable around 4900–5200 kg ha^−1^, showing only minor fluctuations as rainfall increases. However, March rainfall has a much more pronounced effect: at low rainfall levels, the yield drops significantly (around 2200 kg ha^−1^), but it increases steeply with more rainfall, eventually matching the yields seen in other months. This indicates that rainfall in March is particularly critical for achieving optimal meadow yield.

**Figure 7:**
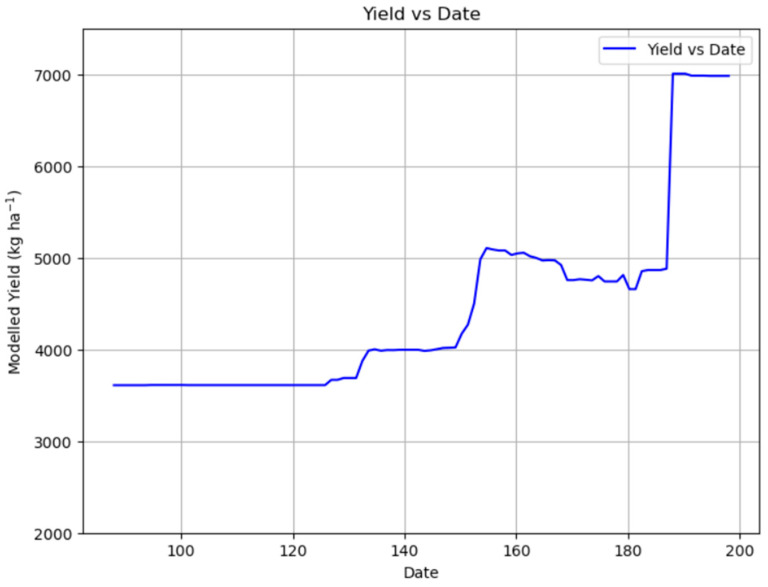
Partial dependence plot showing the impact of the cutting date on yield.

Figure 7 shows the evolution of the meadow yield (in kg ha^−1^) as a function of the cutting date (expressed as the day of the year). Initially, yield remains stable at around 3600 kg ha^−1^ until about day 125, after which it begins to gradually increase. A sharp rise occurs between days 140 and 155, reaching around 5200 kg ha^−1^. Following this peak, the yield slightly declines and stabilizes with minor fluctuations. A significant jump in yield is observed around day 190, where it sharply increases to approximately 7000 kg ha^−1^. This indicates that delaying the cutting date significantly boosts yield, with the highest productivity achieved in later cuts.

**Figure 8:**
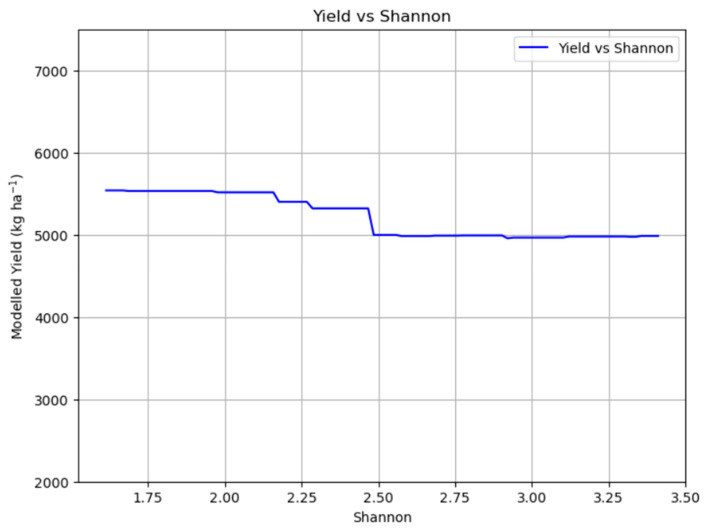
Partial dependence plot showing the impact of biodiversity on yield.

Figure 8 shows the relationship between meadow yield and biodiversity. As biodiversity increases, there is a slight decline in yield, but the reduction is relatively small in percentage terms (10%). Even with higher Shannon index values, the decrease in productivity remains modest, suggesting that promoting biodiversity in meadows does not lead to a substantial loss in yield and may be a viable strategy for balancing agricultural production with ecological sustainability.

#### 2.3.2. Protein Model Variable Impact

The protein model revealed that the most influential factors affecting forage quality are monthly minimum temperatures, the timing of the cut, and biodiversity levels, as indicated by the Shannon index. As a result, these key variables will be examined in detail in the following analysis to assess their specific effects on hay meadow protein content.

**Figure 9:**
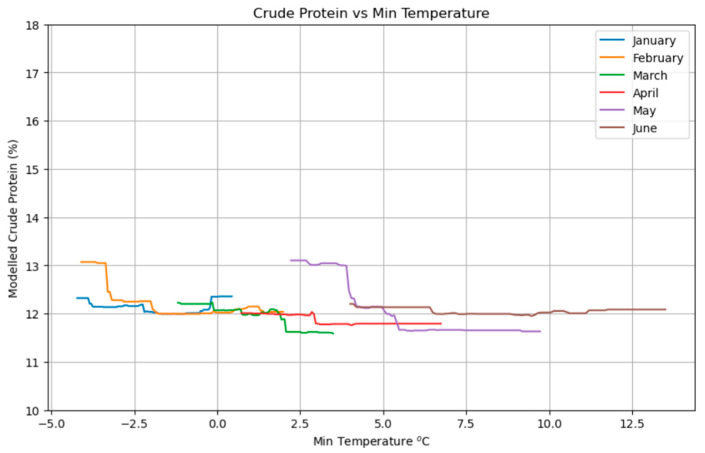
Partial dependence plot showing the impact of monthly minimum temperature on protein content.

Figure 9 displays the relationship between the crude protein (%) of green mass from meadows and the monthly minimum temperatures from January to June. Overall, there is a clear trend indicating that protein content tends to increase as minimum temperatures decrease. For most months, higher protein percentages are observed at lower temperatures, especially noticeable in February and May, where protein levels exceed 13% and 12.8%, respectively, at the coldest values. As temperatures rise, the protein content generally declines, dropping to below 12% in several cases. This suggests that colder conditions may contribute to higher nutritional quality in terms of protein content in forage.

**Figure 10:**
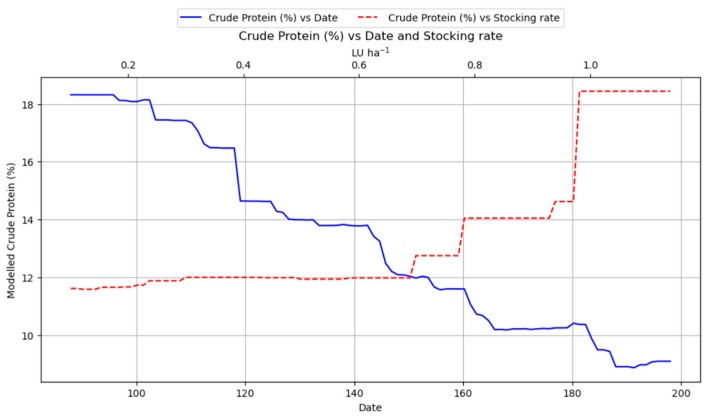
Partial dependence plot showing the impact of cutting date (lower x axis) and stocking rate (upper x axis) on protein content.

Figure 10 depicts how the protein content (%) in meadows evolves in relation to two key factors: the cutting date (expressed as the day of the year) and the livestock load (measured in livestock units, LU ha^−1^). On one side, the figure shows that protein content declines steadily as the cutting date is delayed. In other words, earlier cuts yield higher protein levels, whereas later cuts lead to markedly lower protein percentages. This clear trend emphasizes the importance of early harvesting if one aims to maximize the nutritional quality of the forage. Simultaneously, Figure 10 also illustrates the influence of grazing pressure on protein content. The data indicates that as livestock load increases, the protein content tends to rise gradually, peaking around 1 LU ha^−1^.

**Figure 11:**
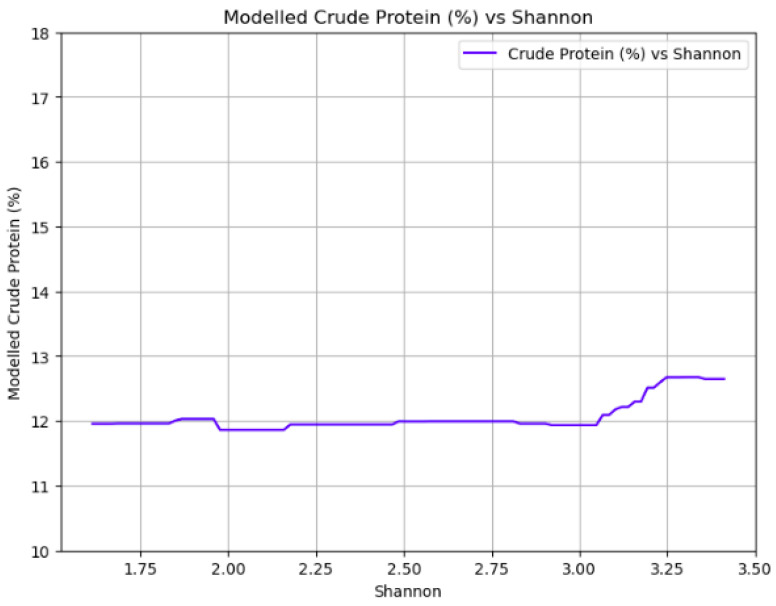
Partial dependence plot showing the impact of biodiversity on protein content.

Figure 11 illustrates the relationship between protein content (%) in meadows and biodiversity, measured by the Shannon index. The protein content remains relatively stable across the increasing biodiversity levels until the Shannon index reaches a value of 3. At that point, there is a sharp increase of about 6% in protein content, suggesting that very high biodiversity levels may significantly enhance the nutritional quality of the hay.

#### 2.3.3. RFV Model Variable Impact

In the RFV model, monthly minimum temperatures, cutting date, and biodiversity were identified as the variables with the greatest impact on forage quality. Therefore, these factors will be the focus of the following analysis to better understand their influence on forage meadow nutritional value.

**Figure 12:**
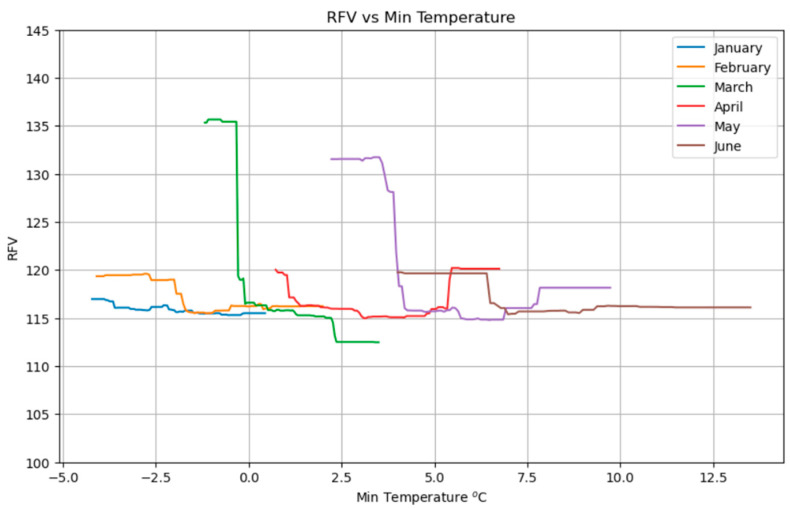
Partial dependence plot showing the impact of minimum temperature on RFVs.

Figure 12 shows the evolution of the RFV in relation to monthly minimum temperatures from January to June. Like the trend observed for protein content, RFV increases as minimum temperatures decrease, indicating improved forage quality under colder conditions. This effect is particularly pronounced for the months of March and May, where lower minimum temperatures are clearly associated with higher RFVs.

**Figure 13:**
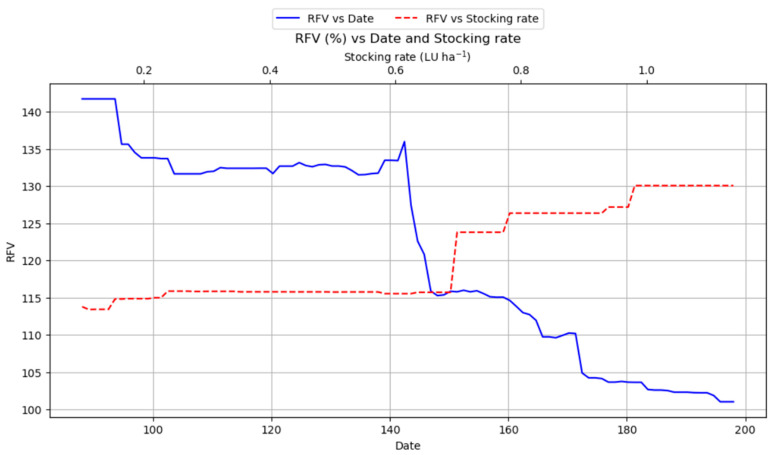
Partial dependence plot showing the impact of cutting date (lower × axis) and stocking rate (upper × axis) on RFVs.

Figure 13 presents two complementary perspectives on forage quality by illustrating the behavior of RFVs under different management practices. On one hand, the figure depicts the evolution of the RFV with respect to the cutting date. Up to around day 140, RFV remains relatively constant; however, beyond that point, there is a dramatic drop (from roughly 135 to 115), followed by a continued gradual decline as the cutting date is delayed. This pronounced pattern underscores the critical role of harvest timing: earlier cuts are strongly associated with higher RFV, hence, superior forage quality. On the other hand, Figure 13 shows the relationship between RFV and livestock density, expressed in livestock units (LUs). RFV is stable at lower grazing pressures (up to about 0.7 LU); however, beyond this threshold, RFV increases steadily, reaching its maximum at 1 LU. This trend closely mirrors the pattern observed for protein content, suggesting that moderate grazing pressure may enhance forage quality by positively influencing both nutritional value and digestibility.

**Figure 14:**
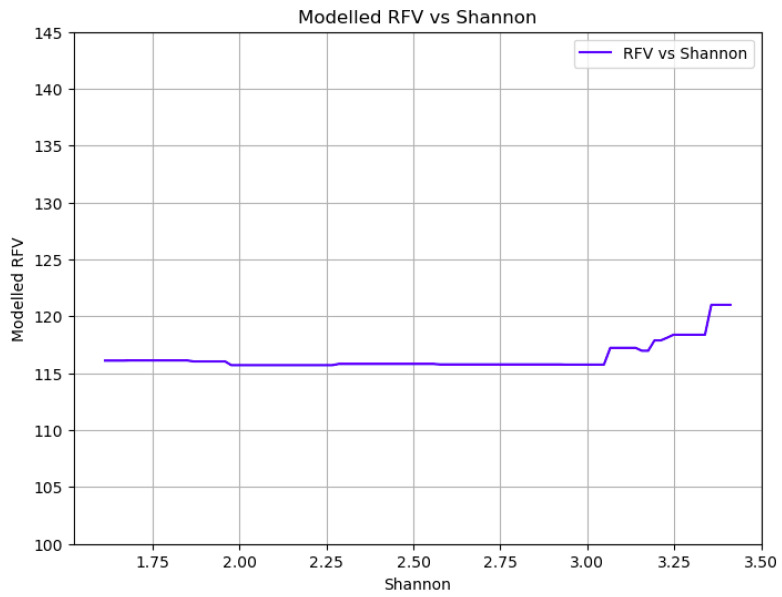
Partial dependence plot showing the impact of livestock load on RFV.

Figure 14 illustrates the relationship between the RFV and biodiversity, measured using the Shannon index. The RFV remains relatively stable across increasing biodiversity levels until the index reaches a value of three, where a moderate increase in RFV is observed. Although this pattern is similar to that seen for protein content, the rise in RFV is less pronounced, suggesting that while high biodiversity may enhance forage quality, its impact on digestibility and intake potential is more limited compared to its effect on nutritional composition.

## 3. Discussion

Figure 1 clearly demonstrates that hay meadows constitute an important reservoir of biodiversity, a fact that underscores the need for their conservation. The data reveal that these agroecosystems not only harbor a rich variety of species [14]. Given that the sustainability of these meadows is intrinsically linked to the value they add to the livestock systems managed by local farmers, it becomes vitally important to refine management practices to safeguard their biodiversity while sustaining robust forage yields [32]. In this context, we employed Random Forest models to assess the impact of various parameters and to track their evolution under different environmental and management conditions, providing critical insights into the dynamic interplay between biodiversity, productivity, climate, and agricultural practices.

The Random Forest regression approach demonstrated remarkable predictive accuracy in this study, explaining nearly 80% of the variance in yield (R^2^ = 0.8018), 79% in protein content (R^2^ = 0.7855), and 72% in RFV (R^2^ = 0.718). These performance metrics are highly competitive and, in some cases, exceed those reported in other agricultural prediction studies utilizing machine learning [29,33]. The ensemble method’s inherent ability to capture complex, non-linear relationships among predictors, coupled with its robustness ensured through rigorous cross-validation, represents a significant advantage in ecological modeling. This is particularly pertinent in natural systems where interactions among environmental and management factors are frequently non-additive and intricate [34]. Furthermore, a key strength of the Random Forest model is its capability to estimate feature importance, which was instrumental in identifying the primary drivers (e.g., March rain for yield, cutting date for quality) for different forage outcomes. This provides invaluable insights into the underlying ecological processes and directly informs targeted management interventions. Additionally, the Random Forest model is known for its robustness to outliers and its ability to handle missing values, making it a highly suitable tool for real-world ecological datasets that often present such complexities [35].

Despite its numerous strengths, the Random Forest approach does present certain limitations. Computationally, larger forests can be resource-intensive, potentially increasing running time and storage requirements. While this was likely manageable for the current dataset, it remains a consideration for very large-scale or real-time applications. Another common critique is that Random Forest models can sometimes be perceived as “black box” algorithms due to their inherent complexity, which can obscure the precise decision-making logic of individual trees [36]. However, the explicit output of feature importance, as utilized in this study, partially mitigates this by clearly highlighting the most influential variables. Lastly, for regression tasks, Random Forest may not predict beyond the range of training data and can occasionally overfit particularly noisy datasets. Given the robust cross-validation applied and the high R^2^ values achieved, these issues were likely not significant concerns in the context of this study. The successful and accurate application of Random Forest across multiple forage traits (yield, protein, RFV) reinforces the paradigm shift towards machine learning as a foundational and indispensable tool in agricultural and ecological research, enabling more sophisticated modeling of complex biological systems. Machine learning methods have been widely adopted for crop yield prediction, often outperforming traditional statistical models. The ability of machine learning models to process complex data and account for high variability makes them particularly suitable for forage management, forming the basis for AI systems that provide real-time recommendations [37]. This success signifies a broader transition in how ecological and agricultural systems are understood and managed. Machine learning, with its capacity to process high-dimensional, non-linear data, offers a powerful alternative to traditional statistical models, paving the way for more precise predictions and enabling the development of advanced decision support systems for sustainable land management [33,38].

As depicted in Figure 5, the analysis reveals that climatic factors exert a substantially greater influence on meadow yield and feed quality compared to other variables considered in this study. This highlights the critical role of climate in shaping the productivity and nutritional value of mountain meadows.

A pivotal finding of this study is the exceptional importance of March rainfall in determining meadow biomass yield. The Random Forest model assigned March rain an importance value of 0.430 in the yield prediction model (Table 2). This high value unequivocally indicates that early spring precipitation is the primary climatic driver of biomass accumulation. This is likely due to its crucial role in supplying essential moisture needed during the critical initial growth stages of meadow plants, thereby boosting plant vigor and leading to enhanced yield outcomes [39].

The disproportionate influence of March rainfall on yield (Figure 6), compared to rainfall in other months, highlights that the timing and seasonal distribution of precipitation are more critical for meadow productivity in temperate grasslands than the total annual rainfall amount [40]. High intra-annual precipitation variability has been shown to decrease aboveground productivity in mesic temperate grasslands, particularly when coinciding with low annual precipitation amounts [12,41]. This suggests that future climate change, which is predicted to increase intra-annual precipitation variability and the frequency of extreme rainfall events, could significantly impact yields even if total annual precipitation remains constant [42]. This necessitates a focus on early-season moisture management strategies to mitigate potential productivity losses.

The consistent inverse relationship between minimum temperatures and forage quality (protein content and RFV) suggests that colder conditions during key growth periods may promote higher nutritional value (Figure 9 and Figure 12). This could occur by slowing plant maturation or reducing metabolic losses, allowing for greater accumulation of non-structural carbohydrates and proteins before plants enter reproductive stages where structural components increase and digestibility/protein content decline [43]. This implies that projected warmer winters and springs under climate change could lead to a systemic decline in meadow forage quality, impacting livestock nutrition. If minimum temperatures continue to rise due to climate change, even if biomass yield is maintained, the nutritional quality of forage could systematically decrease, posing a long-term challenge for livestock production and requiring adaptation strategies to mitigate the decline in feed value [44].

The time of harvest, or cutting date, emerged as a dominant factor in predicting forage quality, particularly for protein content (importance value of 0.366) and RFV (importance value of 0.344). The results consistently show a clear decline in both protein content and RFV as the cutting date progresses. Early cuttings are strongly associated with higher protein levels and RFV, while later cuts result in significantly lower values [13,24,45]. This trend is well-established in agronomic literature, where forage quality, including crude protein and RFV, is known to decrease with advancing plant maturity due to an increasing stem-to-leaf ratio and a decline in mineral content [46,47].

In contrast, while the cutting date was still an influential factor for yield predictions (importance value of 0.194), its importance was noticeably smaller compared to March rainfall. Figure 7 illustrates that delaying the cutting date generally boosts total biomass yield, with a significant increase observed around day 190, where it sharply increases to approximately 7000 kg/ha (Figure 7). This graphical representation clearly demonstrates a fundamental trade-off: higher yields are achieved with later cuts, but at the direct expense of forage quality (Figure 10 and Figure 13). This inverse relationship between cutting date and forage quality (protein, RFV) versus its positive relationship with total biomass yield presents an inherent management dilemma [13].

The study revealed a positive relationship between livestock load and forage quality (Figure 10 and Figure 13). Protein content and RFV both increased progressively with higher livestock density, reaching their peak values at approximately 1 LU ha^−1^. This trend suggests that moderate grazing pressure may contribute to enhancing the nutritional quality of the forage. The positive effect of moderate livestock load on protein and RFV indicates an optimal grazing intensity that can enhance forage quality [48]. Moderate levels of disturbance have been suggested as optimal grassland management strategies for root production in alpine meadows [49]. This supports the implementation of managed grazing systems like rotational grazing, which can improve forage utilization, allow for higher stocking rates, enhance soil health, and prevent overgrazing by allowing grasses to recover between grazing cycles [50]. Moderate grazing, possible through mechanisms like stimulating regrowth of younger, more nutritious plant parts, enhancing nutrient cycling, or maintaining a more vegetative state, can improve quality [51]. This reinforces the value of adaptive grazing management, which can maintain productivity and quality while preventing the degradation associated with overgrazing [52,53].

While the variable importance table did not explicitly rank fertilization rates for protein or RFV, the initial results acknowledged fertilization (N, P, K kg ha^−1^ rates) as a key management input influencing meadow outcome (Table 2). Some studies corroborate that fertilization, particularly with nitrogen and phosphorus, can significantly increase forage yield and can potentially improve crude protein [22,54]. However, it is crucial to note that long-term studies on NPK fertilization have shown that combined additions of N and P (with or without K) can have a strong negative impact on species richness in grasslands [55,56].

Looking at the relation between yield and biodiversity, the results indicate that as biodiversity increases, there is a slight decline in yield, but this reduction is relatively small, approximately 10% (Figure 8). This suggests that promoting biodiversity in meadows does not lead to a substantial loss in overall productivity. The modest trade-off between increased biodiversity and a small reduction in yield is a crucial finding for sustainable agriculture, demonstrating that maintaining or enhancing meadow biodiversity is a viable strategy for balancing agricultural production with ecological sustainability [4,26]. On the other hand, the relationship between biodiversity and forage quality revealed interesting patterns. Protein content remained relatively stable across increasing biodiversity levels until the Shannon index reached a value of 3 (Figure 11 and Figure 14). At this threshold, a sharp increase of about 6% in protein content was observed. RFV exhibited a similar trend, remaining stable until a Shannon index of 3, where a moderate increase in RFV was observed, although this rise was less pronounced compared to protein content. The observed threshold effect, where significant improvements in protein content and RFV only occur beyond a certain level of biodiversity (Shannon index of 3), suggests that a critical mass of species richness and evenness is necessary to unlock the full nutritional benefits of diverse meadow communities. This non-linear response implies that below a certain diversity threshold, the benefits of species interactions (e.g., complementary resource use, nitrogen fixation by legumes, altered plant–plant interactions) are not fully realized.

Crucially, our approach allowed us to clearly identify the interaction between management, climate, and meadow characteristics in yield and quality, using machine learning techniques that are not extensively described in the existing scientific literature.

## 4. Materials and Methods

### 4.1. Study Area and Experimental Design

The research was conducted in the central Spanish Pyrenees (42°30′–42°50′ N, 0°10′–0°40′ E), a mountainous region characterized by diverse topography and varying altitudes. Fifteen meadows were selected across different farms within the Aragón, Gállego, Ara, Cinca, and Ésera valleys to ensure spatial independence and to represent the range of agricultural management practices prevalent in the area. The study spanned five years, with data collected in 2019, 2020, and 2022 through 2024.

### 4.2. Farmer Surveys

Detailed information on agricultural management practices was obtained through surveys carried out by the farmers managing the 15 selected meadows. These surveys employed a structured questionnaire or interview protocol to ensure consistency in data collection. Information gathered included (i) type of livestock (species and breed), the number of animals, and the duration and timing of their grazing periods, which allowed for the calculation of stocking rate in livestock units (LUs); (ii) type of fertilization (organic manure, inorganic fertilizers, or none) and the rate and frequency of fertilization (in order to obtain the applied units of N, P, and K). For manure or slurry, samples were taken for laboratory analysis to determine their nutrient content; and (iii) other management practices such as mowing frequency (outside experimental cuts).

### 4.3. Vegetation Sampling

In each of the 15 meadows, green samples were taken from 6 plot enclosures (40 cm × 60 cm) at different times, between the 20 May and the 10 July, from the beginning of flowering to the time when the farmer cuts the meadow. A total of 554 samples were collected. At each sampling time, aboveground biomass within each enclosure was harvested at a standardized height of 5 cm using a battery-powered hedge trimmer.

### 4.4. Climatic Data Acquisition

Climatic parameters relevant to the study were obtained from the network of meteorological stations operated by the Agencia Estatal de Meteorología (AEMET). Data from stations 9446, 9789A, 9784P, 9814X, 9838A, 9838B, and 9843A were used, covering the period from 2019 to 2024. The specific climatic variables extracted included daily measurements of minimum, maximum, and average temperature and precipitation from the 1st of January to harvest day.

### 4.5. Flora Inventory and Biodiversity Metrics

Flora inventories were conducted in each sampled meadow using the Braun-Blanquet method. Assessments were performed during the period of peak biomass or flowering to ensure identification of most species. The cover abundance of each plant species within sampling quadrats (size to be specified if smaller than the enclosure) was visually estimated. Taxonomic identification was carried out to the species level. The Shannon diversity index (H) was calculated for each meadow using the following formula:

(1) $$H=-\sum_{i=1}^{S}p_{i}\times lnp_{i}$$

where p_i_ is the proportion of the total cover represented by the i-th species and S the total number of species.

### 4.6. Meadow Type

The 15 sampled meadows were classified into three distinct levels of agricultural intensification: intensive, semi-extensive, and extensive. This classification was based on information from the farmer surveys, considering the rate of fertilization (e.g., kg N ha^−1^ year^−1^), grazing intensity (e.g., livestock units ha^−1^ year^−1^ or grazing days year^−1^), and mowing frequency (outside experimental plots). Specific thresholds for each category should be provided in the results or Supplementary Materials.

### 4.7. Biomass Production and Dry Matter Determination

Fresh biomass samples from each 0.24 m^2^ enclosure were weighed in the field and then stored frozen until processing. In the laboratory, the samples were dried at 65 °C for 48 h to determine field dry matter (DM) proportion. Total DM yield (g m^−2^) was calculated by multiplying fresh weight by DM proportion.

### 4.8. Forage Quality Analysis

Forage quality analysis was performed from the total harvested biomass of each 0.24 m^2^ plot. Laboratory dry matter was determined by oven-drying at 105 °C for 4 h. Nitrogen content (N) was determined using the Kjeldahl method, and crude protein (CP) was calculated as N × 6.25. Relative feed value (RFV) was calculated based on Acid Detergent Fiber (ADF) and Neutral Detergent Fiber (NDF) fractions using the following formula [57]:

(2) $$RFV=\left(88.9-\left(0.779\times ADF\right)\times \left(\frac{120}{NDF}\right)\right)/1.29$$

### 4.9. Statistical Analysis and Modeling

All data analysis and statistical modeling were conducted using the Jupyter Lab environment [58] with Python 3 and the scikit-learn library [59]. Random Forest models were developed to simulate (i) yield, (ii) protein content, and (iii) RFV. Predictor variables included climatic parameters, meadow classification, management practices, and biodiversity indices. The models were validated using k-fold cross-validation, and performance was assessed using R-squared (R^2^), Mean Squared Error (MSE), and Root Mean Squared Error (RMSE) [60,61].

The hyperparameters of the Random Forest models for yield, protein content, and relative feed value (RFV) were optimized using Optuna, an open-source hyperparameter optimization framework. This optimization process resulted in the following characteristics for each model:

- Yield model: n_estimators = 158, max_depth = 14, min_samples_split = 5, min_samples_leaf = 1, random_state = 42
- Protein model: n_estimators = 222, max_depth = 13, min_samples_split = 6, min_samples_leaf = 1, random_state = 42
- RFV model: n_estimators = 198, max_depth = 27, min_samples_split = 5, min_samples_leaf = 1, random_state = 42

## 5. Conclusions

Our Random Forest regression models demonstrated high predictive accuracy for biomass yield (R^2^ = 0.802), protein content (R^2^ = 0.786), and RFV (R^2^ = 0.718), underscoring the utility of machine learning in agricultural research. March rainfall emerged as the paramount climatic driver for biomass yield, while the cutting date proved to be the dominant factor influencing forage quality, particularly protein content and RFV. This highlights a fundamental trade-off where maximizing yield often comes at the expense of nutritional quality, necessitating a nuanced approach to management.

The insights gained from this research offer actionable strategies for sustainable meadow management. Recognizing the critical role of early-season moisture for yield, practices enhancing water retention become crucial. For optimizing forage quality, precise harvest timing is paramount, requiring strategic decisions based on specific livestock needs. While fertilization can boost yield, its application must be carefully balanced against potential negative impacts on biodiversity, suggesting that integrating legumes or adopting moderate grazing pressures could offer more sustainable pathways to enhancing forage quality. The finding that increased biodiversity incurs only a minor yield penalty while potentially enhancing forage quality, particularly at higher diversity levels, strongly advocates for multi-objective management that integrates biodiversity conservation with agricultural production goals.

Looking ahead, continued long-term monitoring is essential to fully grasp the cumulative and legacy effects of climate variability on meadow ecosystems. Future research should integrate climate projection models to develop proactive adaptation strategies and delve deeper into the mechanistic understanding of biodiversity’s impact on forage quality. Further development of AI-driven decision support systems, leveraging advanced machine learning and real-time sensor data, holds immense potential for dynamic and precise meadow management. Ultimately, this study reinforces the imperative for a holistic, ecosystem-based approach to grassland management, balancing productivity with ecological resilience to ensure the sustained provision of forage and vital ecosystem services in a changing climate.

## Figures and Tables

**Table 1:** This table summarizes the results obtained each year. Climatic variables refer to dates from the 1st of January to harvest day.

		2019	2020	2022	2023	2024
January	Average temperature °C	2.89	3.58	3.61	1.64	3.92
	Maximum temperature °C	7.38	8.22	9.60	6.80	8.74
	Minimum temperature °C	−1.63	−1.06	−2.37	−2.42	−0.86
	Cumulated rain mm	78.15	95.92	33.87	93.06	64.96
February	Average temperature °C	5.40	6.35	5.64	3.43	5.59
	Maximum temperature °C	12.04	12.56	11.69	9.47	10.69
	Minimum temperature °C	−1.24	0.19	−0.41	−2.59	0.47
	Rain mm	48.00	5.42	30.76	42.38	108.14
March	Average temperature °C	6.95	6.16	5.74	7.33	6.25
	Maximum temperature °C	13.46	10.72	9.67	13.46	11.44
	Minimum temperature °C	0.47	1.61	1.81	1.22	1.08
	Rain mm	14.77	176.71	81.60	25.18	228.37
April	Average temperature °C	7.48	8.72	7.78	9.60	8.40
	Maximum temperature °C	12.50	12.82	13.30	16.02	14.26
	Minimum temperature °C	2.49	4.63	2.25	3.20	2.57
	Rain mm	157.89	118.72	111.70	20.78	60.23
May	Average temperature °C	10.12	12.68	13.24	11.16	10.36
	Maximum temperature °C	16.16	18.43	19.56	17.15	15.99
	Minimum temperature °C	4.06	6.94	6.92	5.13	4.78
	Rain mm	87.43	123.40	31.31	70.61	100.35
June	Average temperature °C	15.07	12.91	16.91	14.79	14.27
	Maximum temperature °C	21.78	18.13	23.54	20.18	20.46
	Minimum temperature °C	8.36	7.65	10.29	9.40	8.10
	Rain mm	0.49	137.36	65.91	174.22	91.70
Annual fertilization	N kg ha^−1^	95.20	101.24	99.59	91.57	91.57
	P kg ha^−1^	114.72	137.46	125.47	116.15	116.15
	K kg ha^−1^	71.74	73.68	77.31	70.05	70.05
Annual stocking rate	Livestock Units (LUs ha^−1^)	0.38	0.34	0.36	0.40	0.40
Cutting date	Day of year	178.62	160.17	156.52	153.71	155.79
Yield	kg ha^−1^	1997.31	4862.54	4684.55	2792.58	6273.54
Crude protein	%	12.05	10.24	11.14	14.08	11.94
RFV	RFV units	123.42	107.12	112.71	126.91	115.28

**Table 2:** This table summarizes the importance of each variable for each model. Values >0.1 are highlighted in bold.

		Yield	Protein	RFV
January	Average temperature °C	0.006	0.033	0.052
	Maximum temperature °C	0.018	0.018	0.028
	Minimum temperature °C	0.011	0.012	0.011
	Rain mm	0.013	0.013	0.008
February	Average temperature °C	0.007	0.020	0.025
	Maximum temperature °C	0.008	0.011	0.020
	Minimum temperature °C	0.037	0.021	0.029
	Rain mm	0.016	0.045	0.008
March	Average temperature °C	0.007	0.023	0.008
	Maximum temperature °C	0.041	0.009	0.008
	Minimum temperature °C	0.012	0.083	**0.134**
	Rain mm	**0.430**	0.021	0.008
April	Average temperature °C	0.006	0.012	0.008
	Maximum temperature °C	0.015	0.013	0.018
	Minimum temperature °C	0.016	0.024	0.013
	Rain mm	0.010	0.021	0.014
May	Average temperature °C	0.004	0.017	0.019
	Maximum temperature °C	0.006	0.008	0.023
	Minimum temperature °C	0.006	0.053	0.078
	Rain mm	0.007	0.006	0.007
June	Average temperature °C	0.010	0.009	0.013
	Maximum temperature °C	0.011	0.019	0.020
	Minimum temperature °C	0.006	0.010	0.007
	Rain mm	0.007	0.021	0.005
Fertilization	N kg ha^−1^	0.005	0.003	0.011
	P kg ha^−1^	0.006	0.003	0.011
	K kg ha^−1^	0.020	0.008	0.005
Livestock load	LUs	0.015	0.054	0.040
Cutting date	Day of year	**0.194**	**0.366**	**0.344**
Biodiversity	Shannon Index	0.025	0.040	0.021
Meadow type		0.014	0.003	0.002

## Data Availability

Datasets are available on request from the authors. The raw data supporting the conclusions of this article will be made available by the authors on request.

## Supplementary Materials

The following supporting information can be downloaded at https://www.mdpi.com/article/10.3390/plants14142150/s1, Meadow classification file.

## Abbreviations

The following abbreviations are used in this manuscript:

RFV	Relative Feed Value
LU	Livestock Units
RMSE	Root Mean Square Error
DM	Dry Matter
CP	Crude Protein
ADF	Acid Detergent Fiber
NDF	Neutral Detergent Fiber
